# Supplementary material for: Microtubule assembly by tau impairs endocytosis and neurotransmission via dynamin sequestration in Alzheimer’s disease synapse model
Source: eLife. 2022 Apr 26;11:e73542. doi: 10.7554/eLife.73542 (PMC9071263; doi:10.7554/eLife.73542)
Supplement: Figure 1—figure supplement 1—source data 1. [file elife-73542-fig1-figsupp1-data1.pptx]

## Slide 1
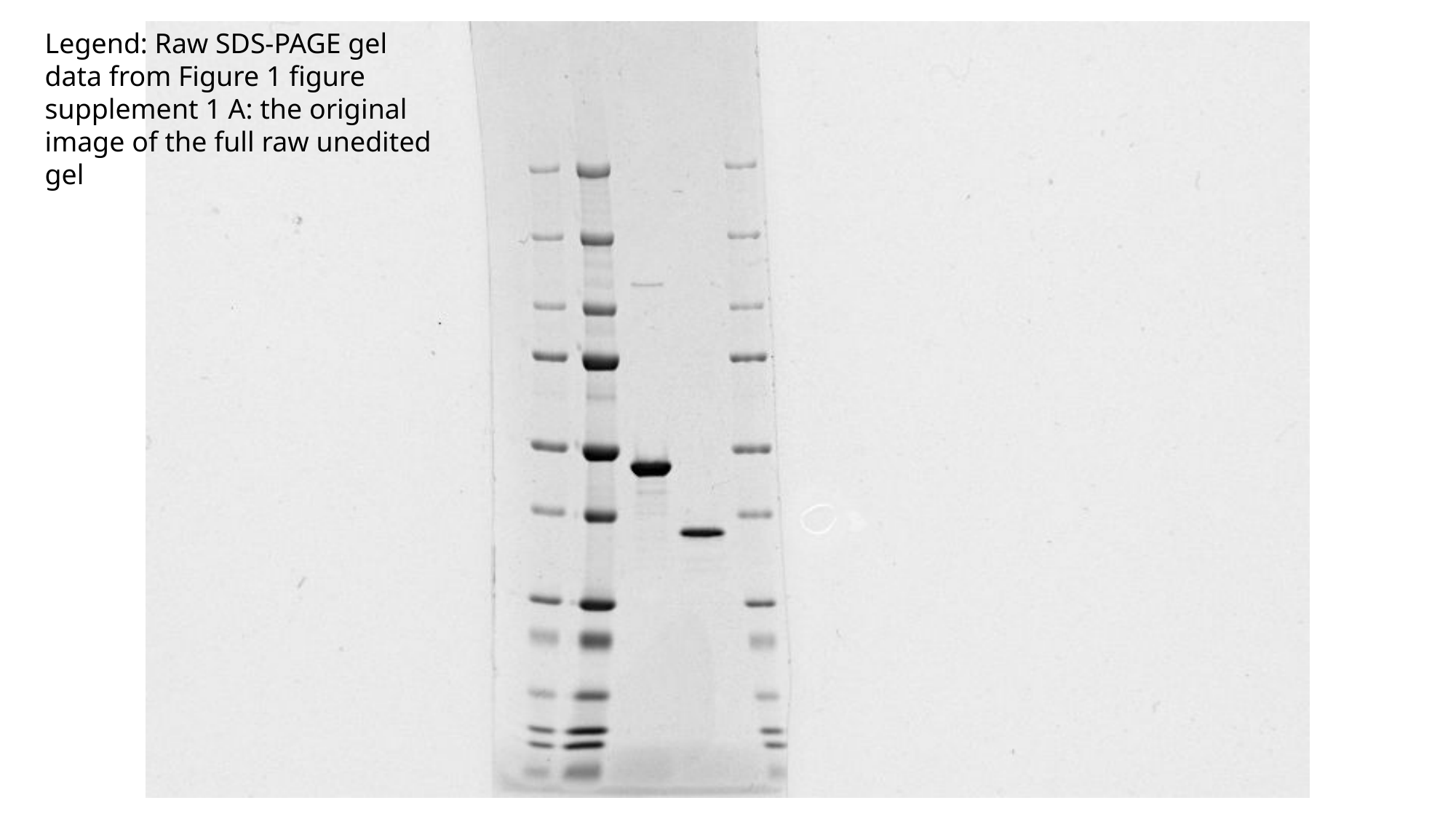

Legend: Raw SDS-PAGE gel data from Figure 1 figure supplement 1 A: the original image of the full raw unedited gel
#

## Slide 2
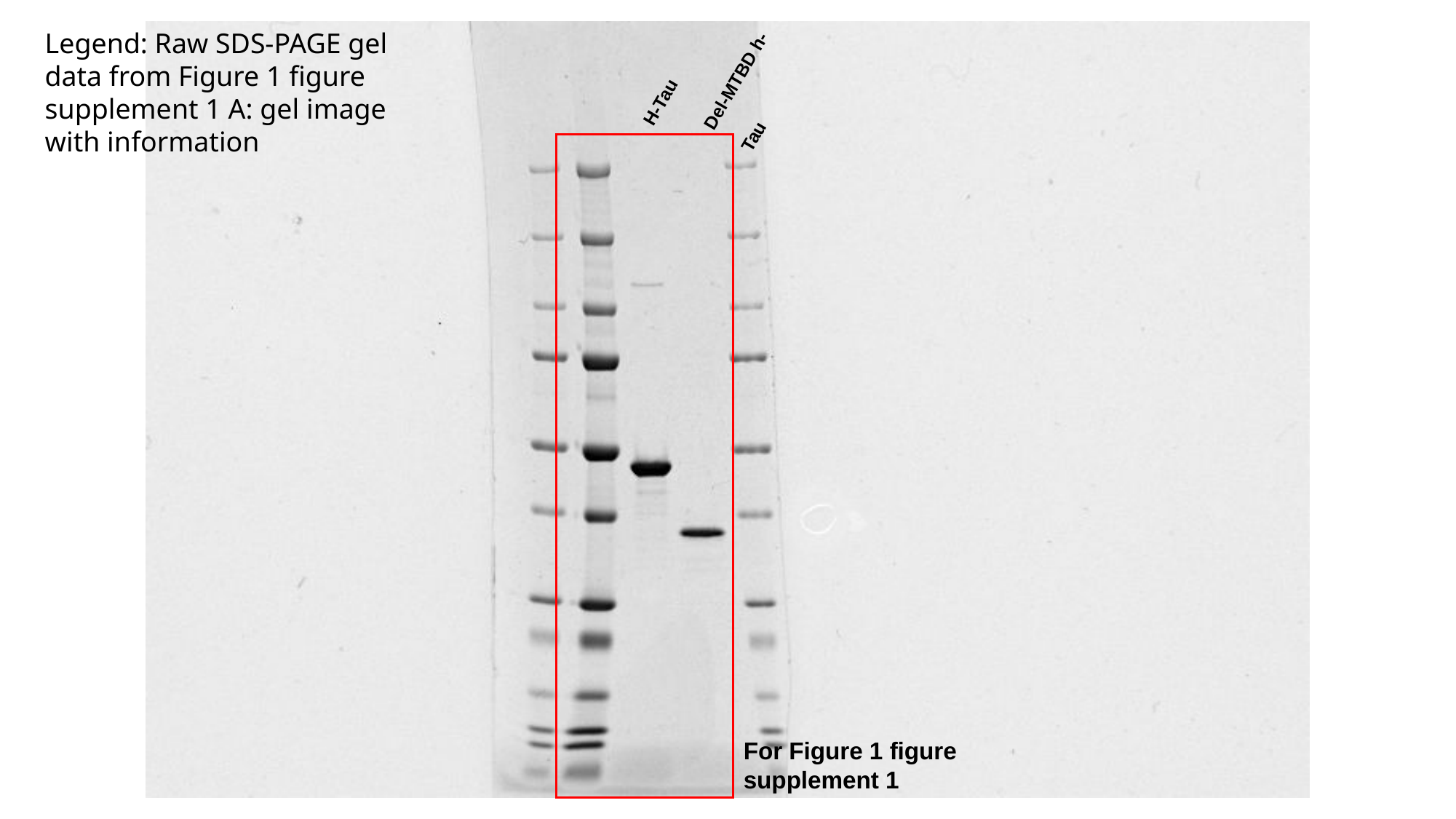

Legend: Raw SDS-PAGE gel data from Figure 1 figure supplement 1 A: gel image with information
Del-MTBD h-Tau
H-Tau
#
For Figure 1 figure supplement 1
