# Supplementary material for: Microtubule assembly by tau impairs endocytosis and neurotransmission via dynamin sequestration in Alzheimer’s disease synapse model
Source: eLife. 2022 Apr 26;11:e73542. doi: 10.7554/eLife.73542 (PMC9071263; doi:10.7554/eLife.73542)
Supplement: Figure 4—figure supplement 1—source data 1. [file elife-73542-fig4-figsupp1-data1.pptx]

## Slide 1
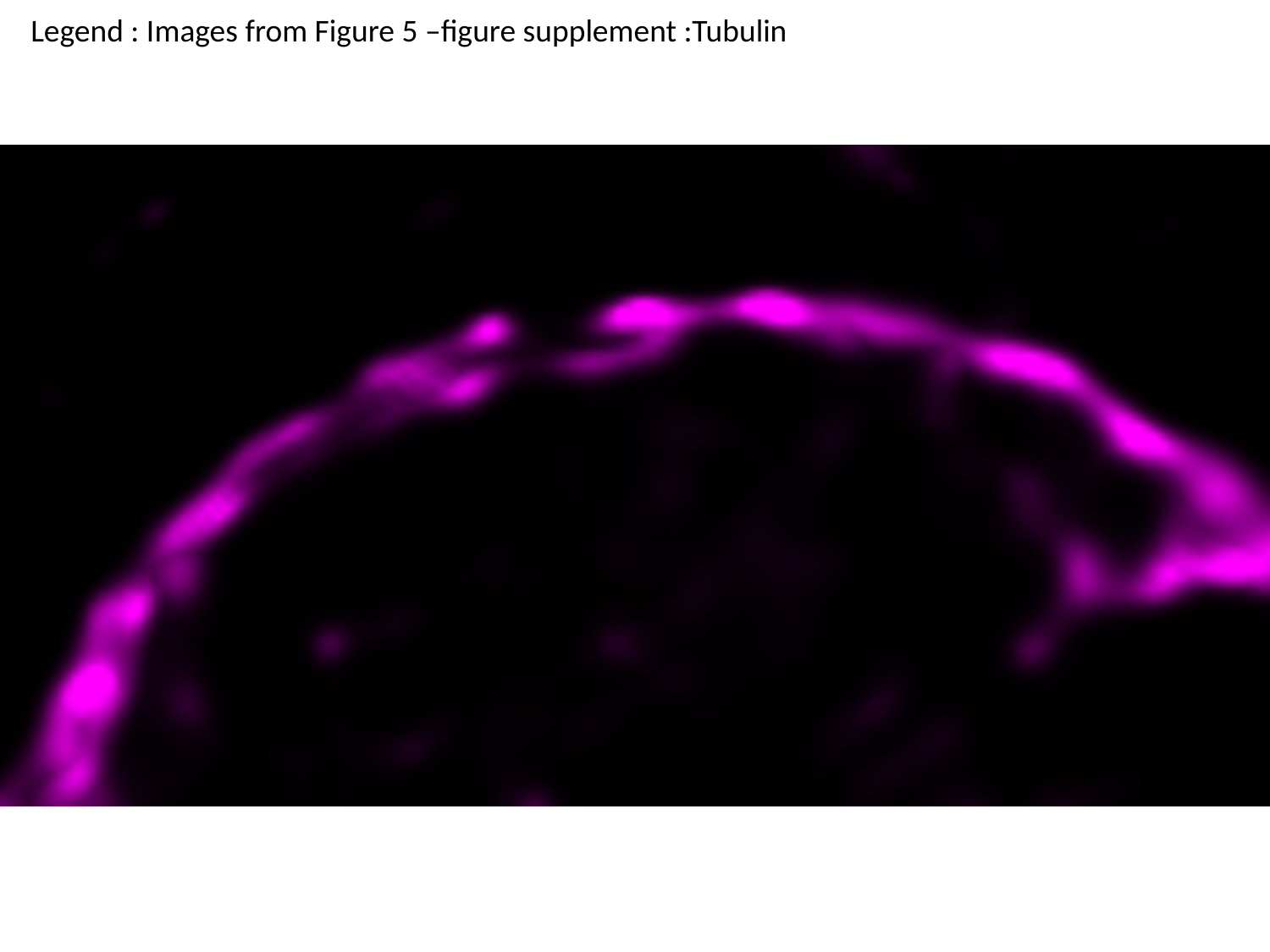

Legend : Images from Figure 5 –figure supplement :Tubulin

## Slide 2
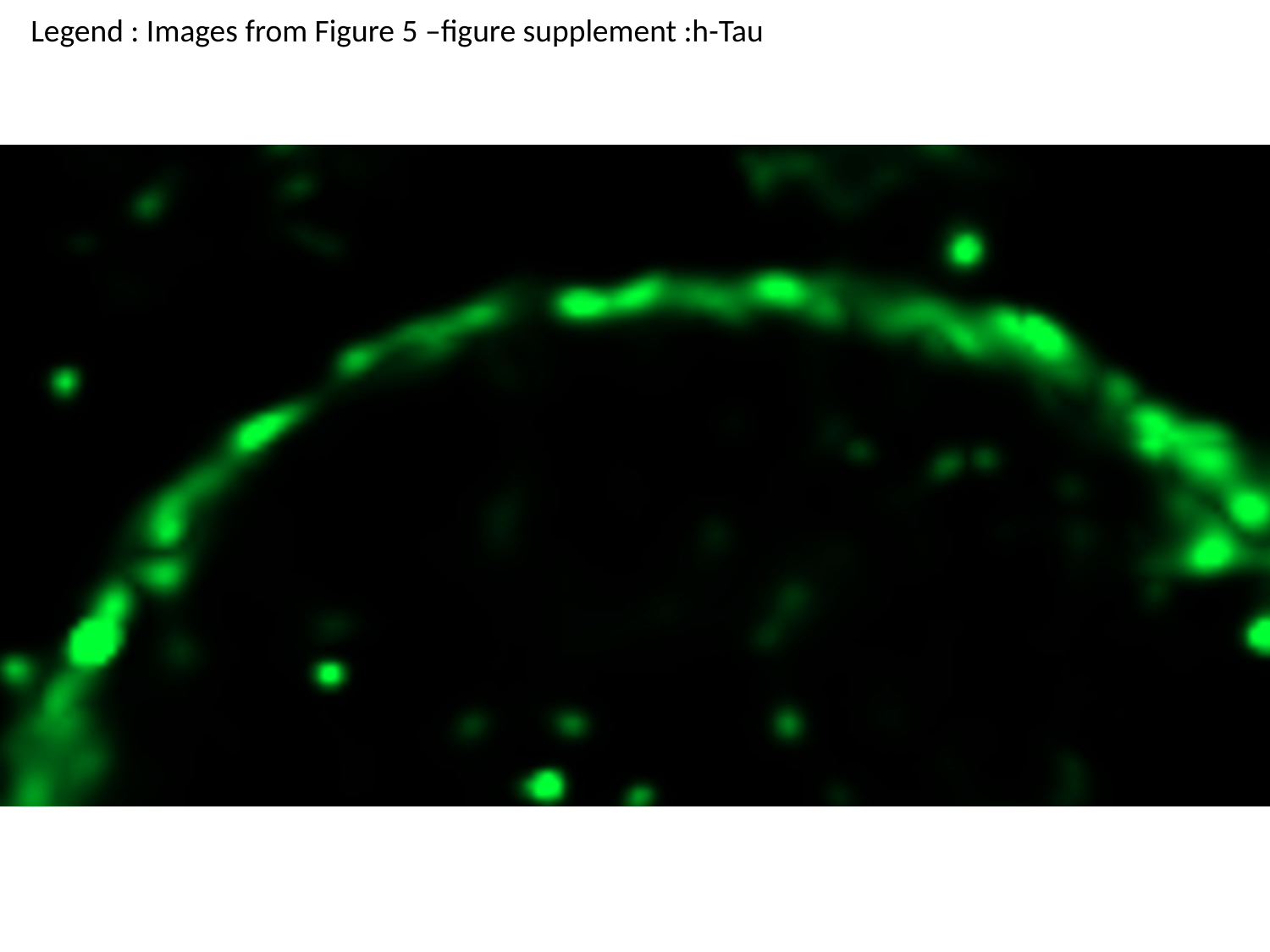

Legend : Images from Figure 5 –figure supplement :h-Tau

## Slide 3
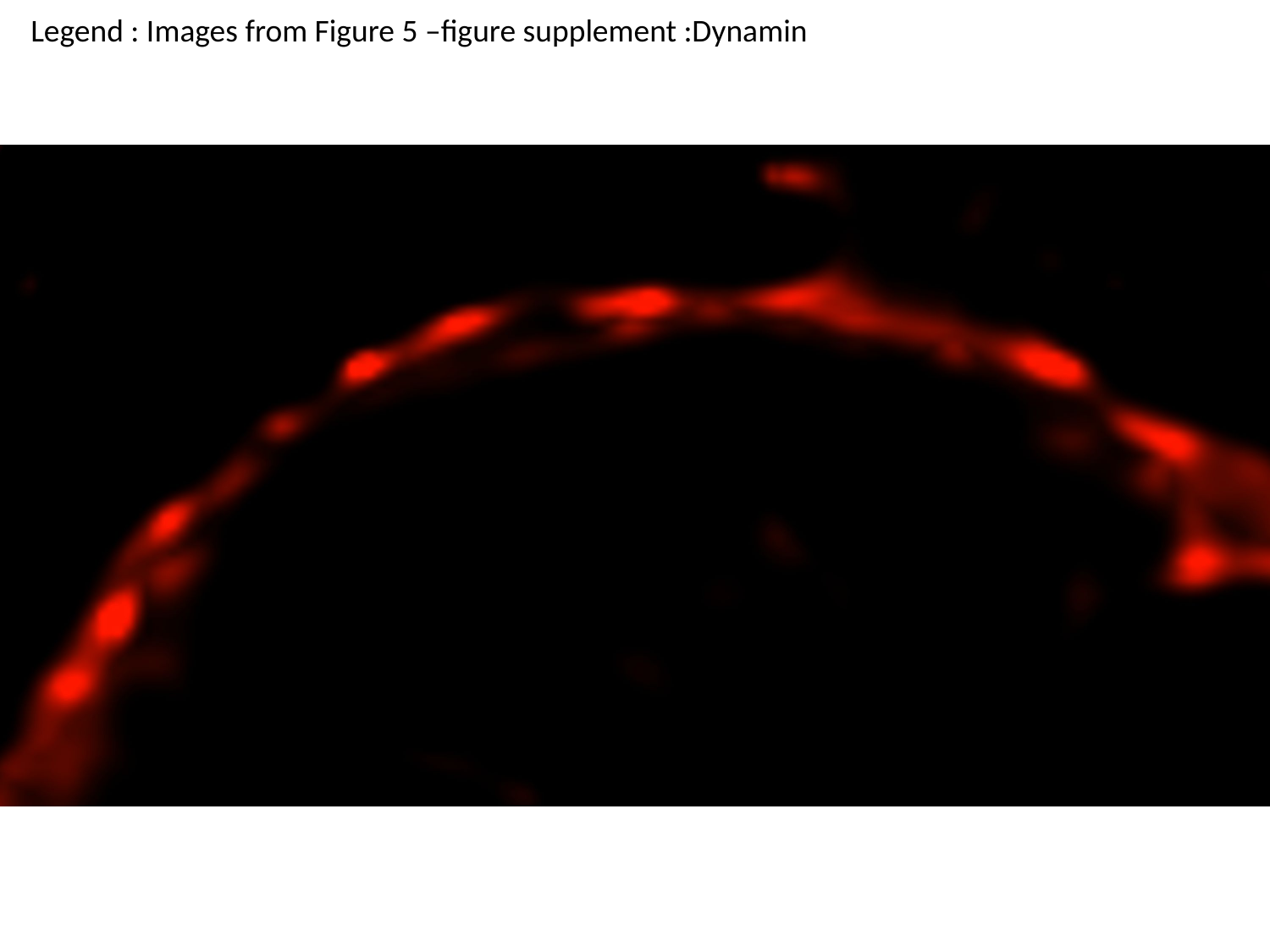

Legend : Images from Figure 5 –figure supplement :Dynamin

## Slide 4
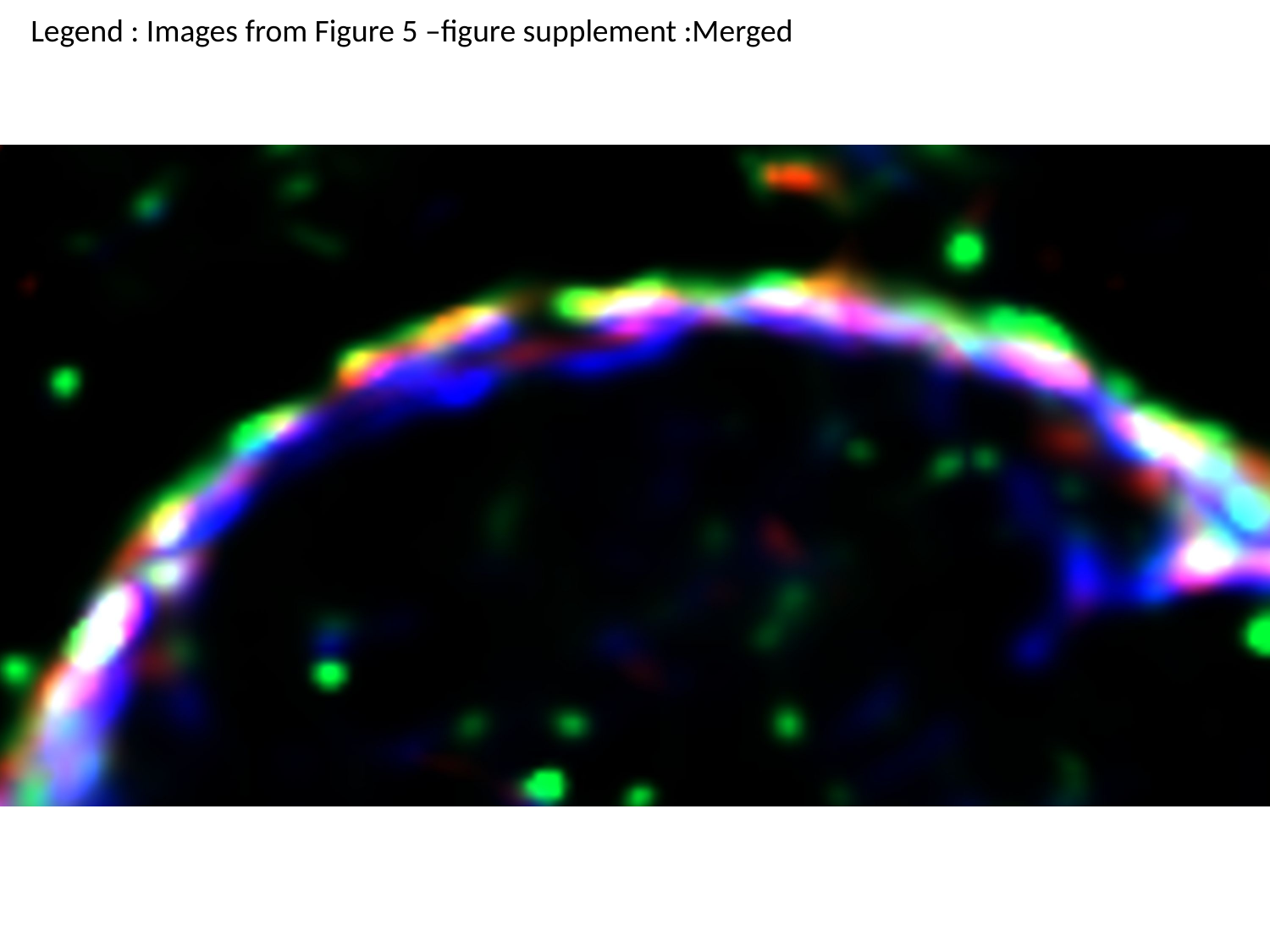

Legend : Images from Figure 5 –figure supplement :Merged
