# Supplementary material for: Microtubule assembly by tau impairs endocytosis and neurotransmission via dynamin sequestration in Alzheimer’s disease synapse model
Source: eLife. 2022 Apr 26;11:e73542. doi: 10.7554/eLife.73542 (PMC9071263; doi:10.7554/eLife.73542)
Supplement: Figure 5—source data 3. [file elife-73542-fig5-data3.pptx]

## Slide 1
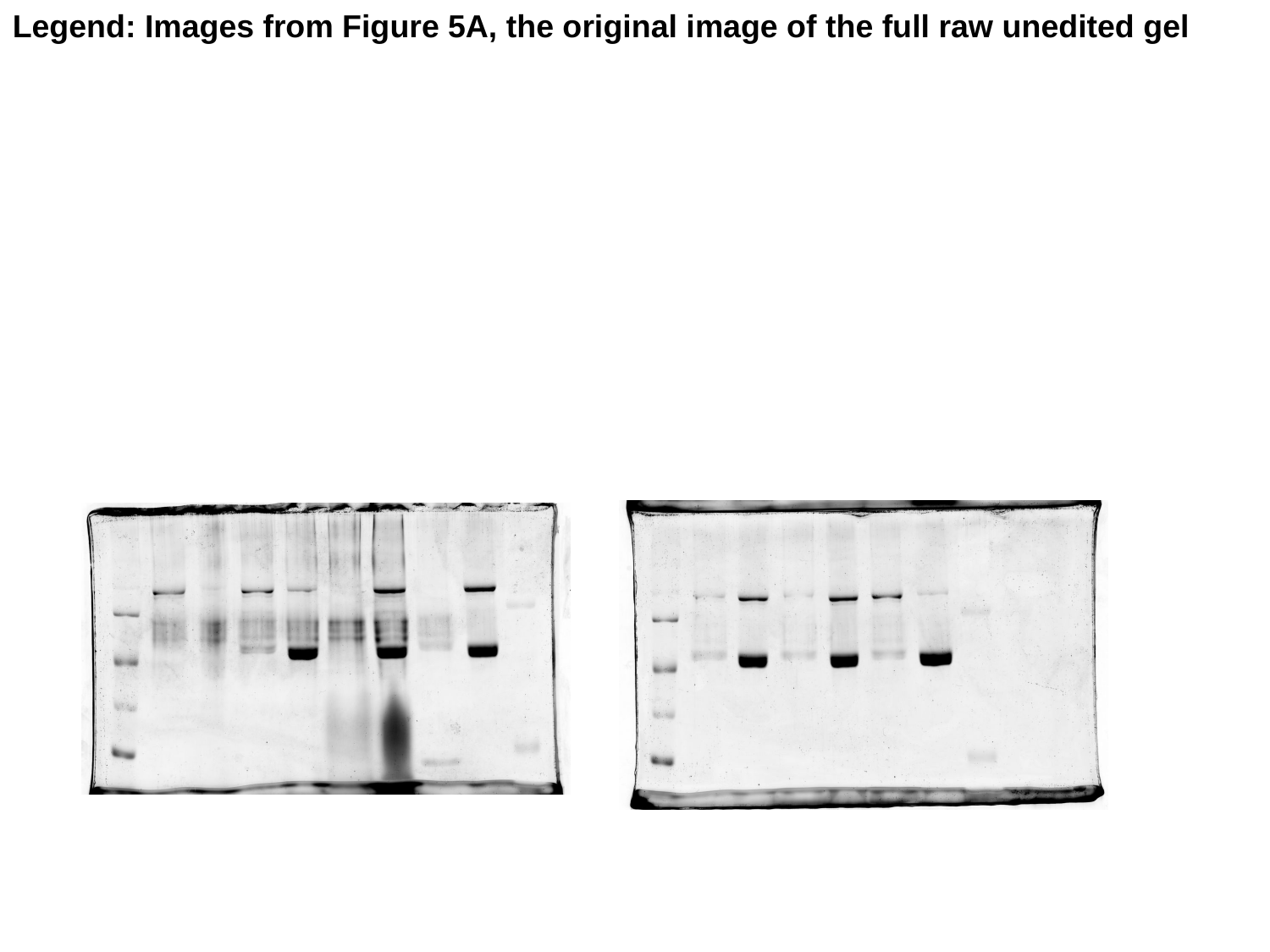

Legend: Images from Figure 5A, the original image of the full raw unedited gel

## Slide 2
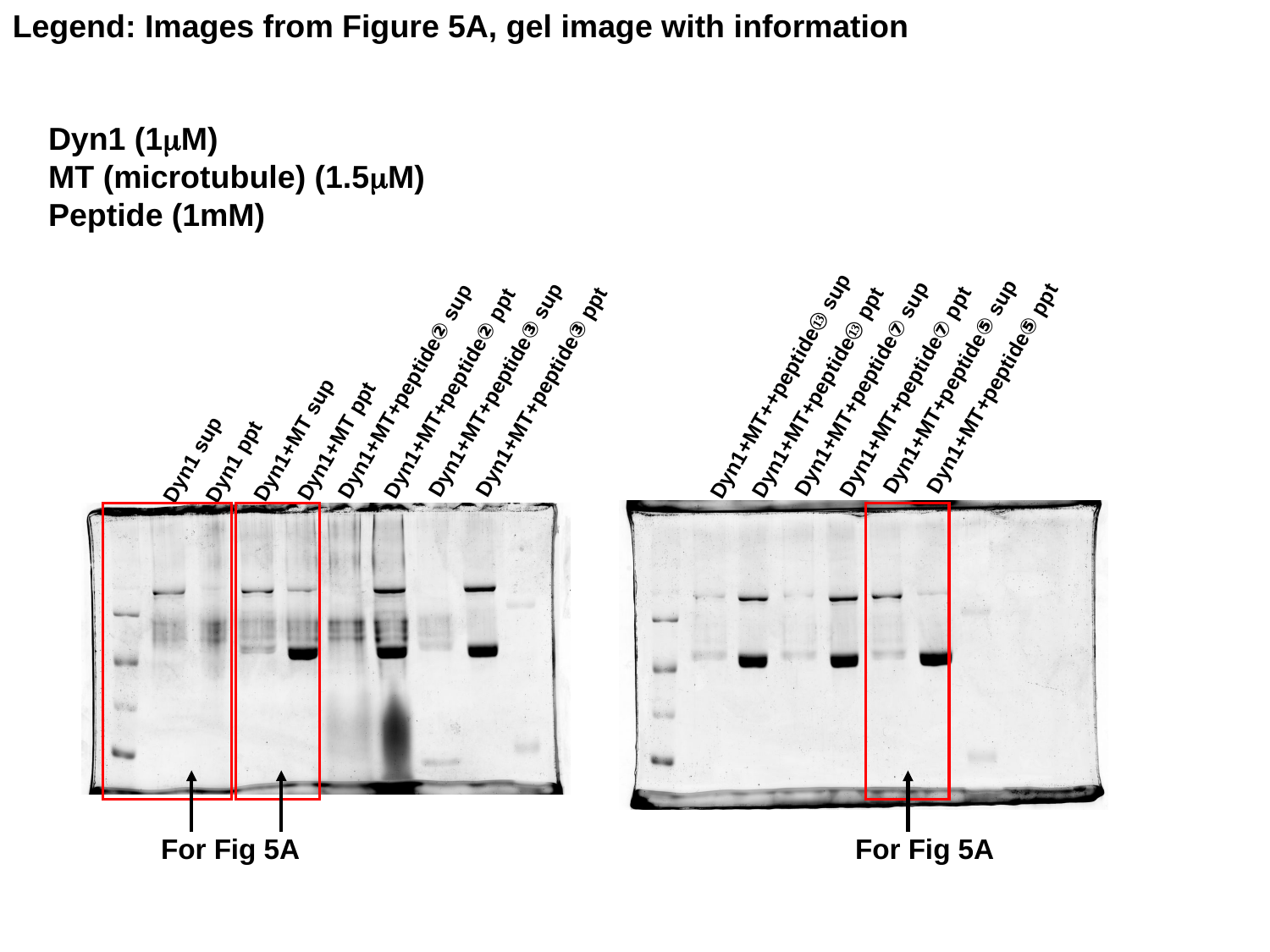

Legend: Images from Figure 5A, gel image with information
Dyn1 (1mM)
MT (microtubule) (1.5mM)
Peptide (1mM)
Dyn1+MT+peptide⑤ ppt
Dyn1+MT+peptide⑤ sup
Dyn1+MT+peptide⑦ sup
Dyn1+MT+peptide⑦ ppt
Dyn1+MT+peptide⑬ ppt
Dyn1+MT++peptide⑬ sup
Dyn1+MT ppt
Dyn1+MT sup
Dyn1+MT+peptide② ppt
Dyn1+MT+peptide③ ppt
Dyn1+MT+peptide② sup
Dyn1+MT+peptide③ sup
 Dyn1 sup
 Dyn1 ppt
For Fig 5A
For Fig 5A
