# Supplementary material for: Microtubule assembly by tau impairs endocytosis and neurotransmission via dynamin sequestration in Alzheimer’s disease synapse model
Source: eLife. 2022 Apr 26;11:e73542. doi: 10.7554/eLife.73542 (PMC9071263; doi:10.7554/eLife.73542)
Supplement: Figure 5—figure supplement 1—source data 1. [file elife-73542-fig5-figsupp1-data1.pptx]

## Slide 1
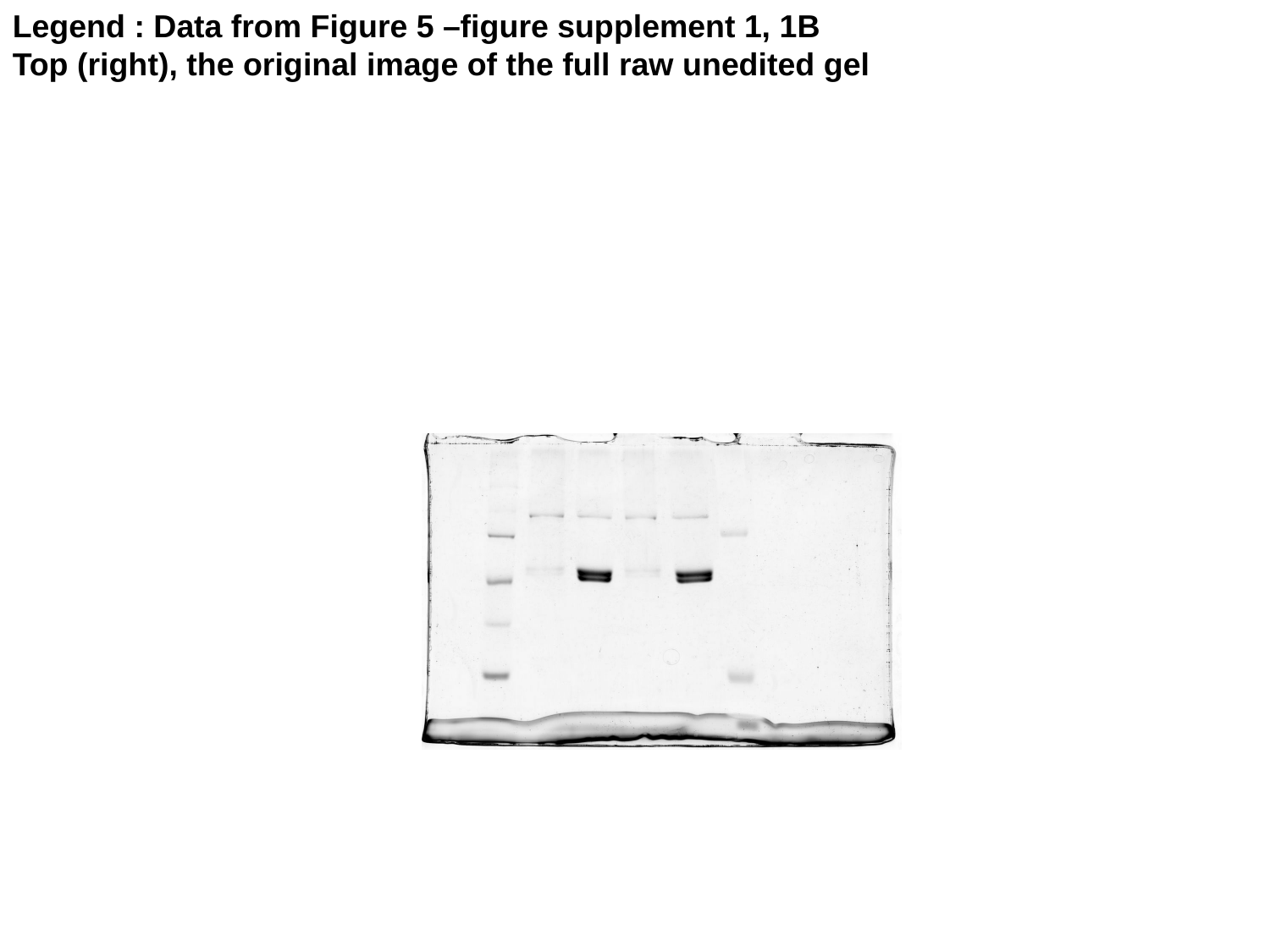

Legend : Data from Figure 5 –figure supplement 1, 1B
Top (right), the original image of the full raw unedited gel

## Slide 2
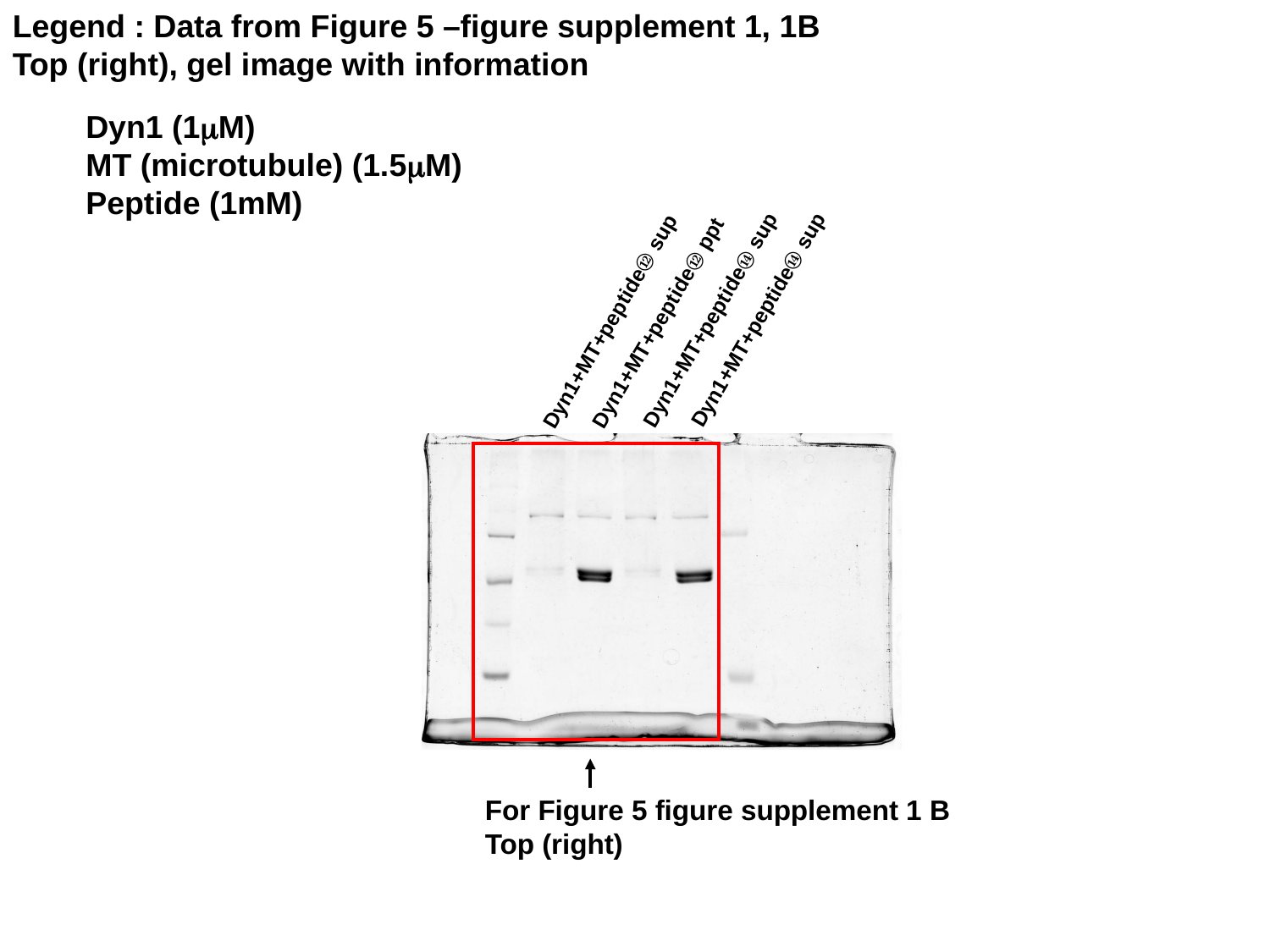

Legend : Data from Figure 5 –figure supplement 1, 1B
Top (right), gel image with information
Dyn1 (1mM)
MT (microtubule) (1.5mM)
Peptide (1mM)
Dyn1+MT+peptide⑫ ppt
Dyn1+MT+peptide⑭ sup
Dyn1+MT+peptide⑭ sup
Dyn1+MT+peptide⑫ sup
For Figure 5 figure supplement 1 B
Top (right)

## Slide 3
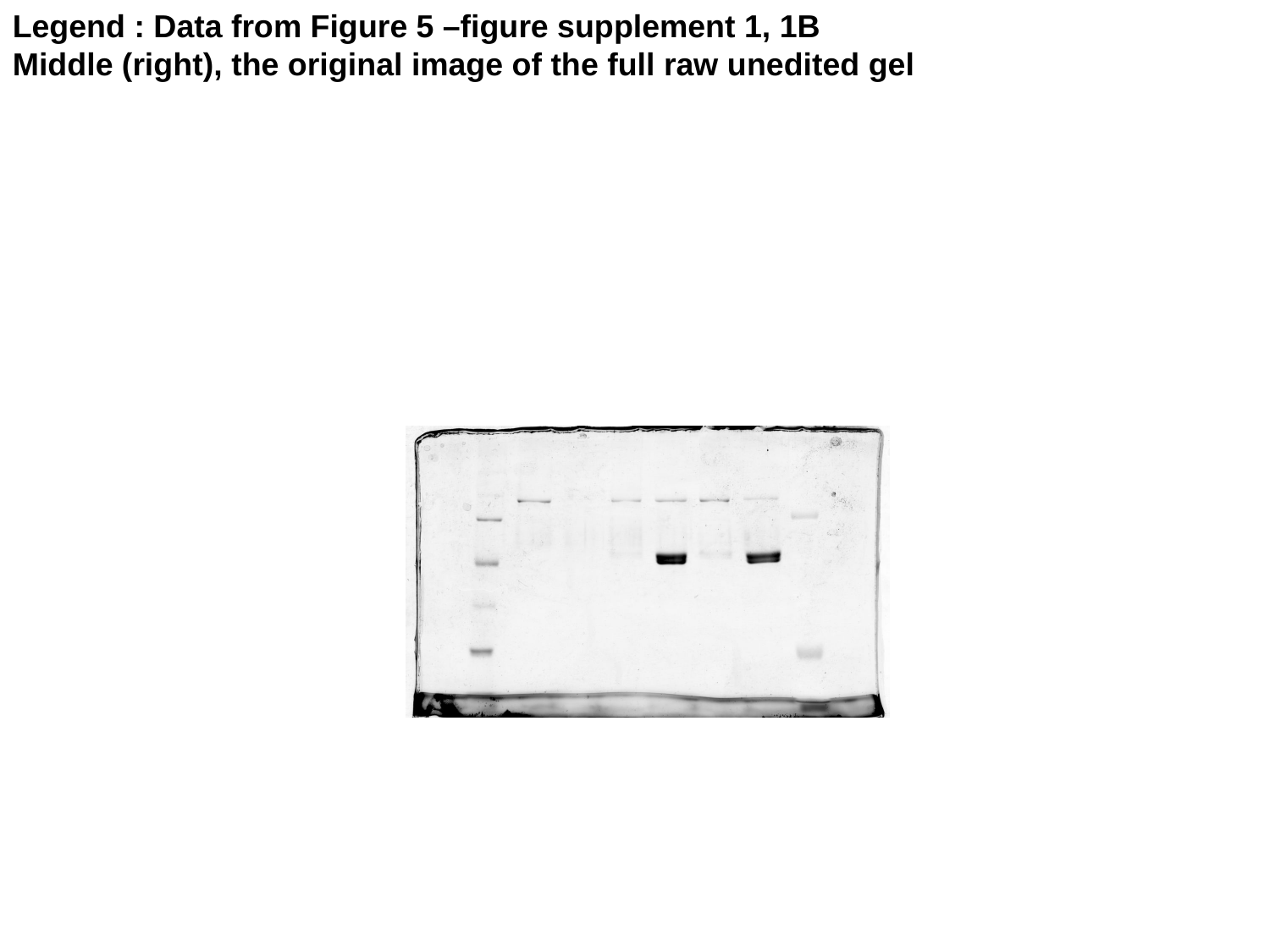

Legend : Data from Figure 5 –figure supplement 1, 1B
Middle (right), the original image of the full raw unedited gel

## Slide 4
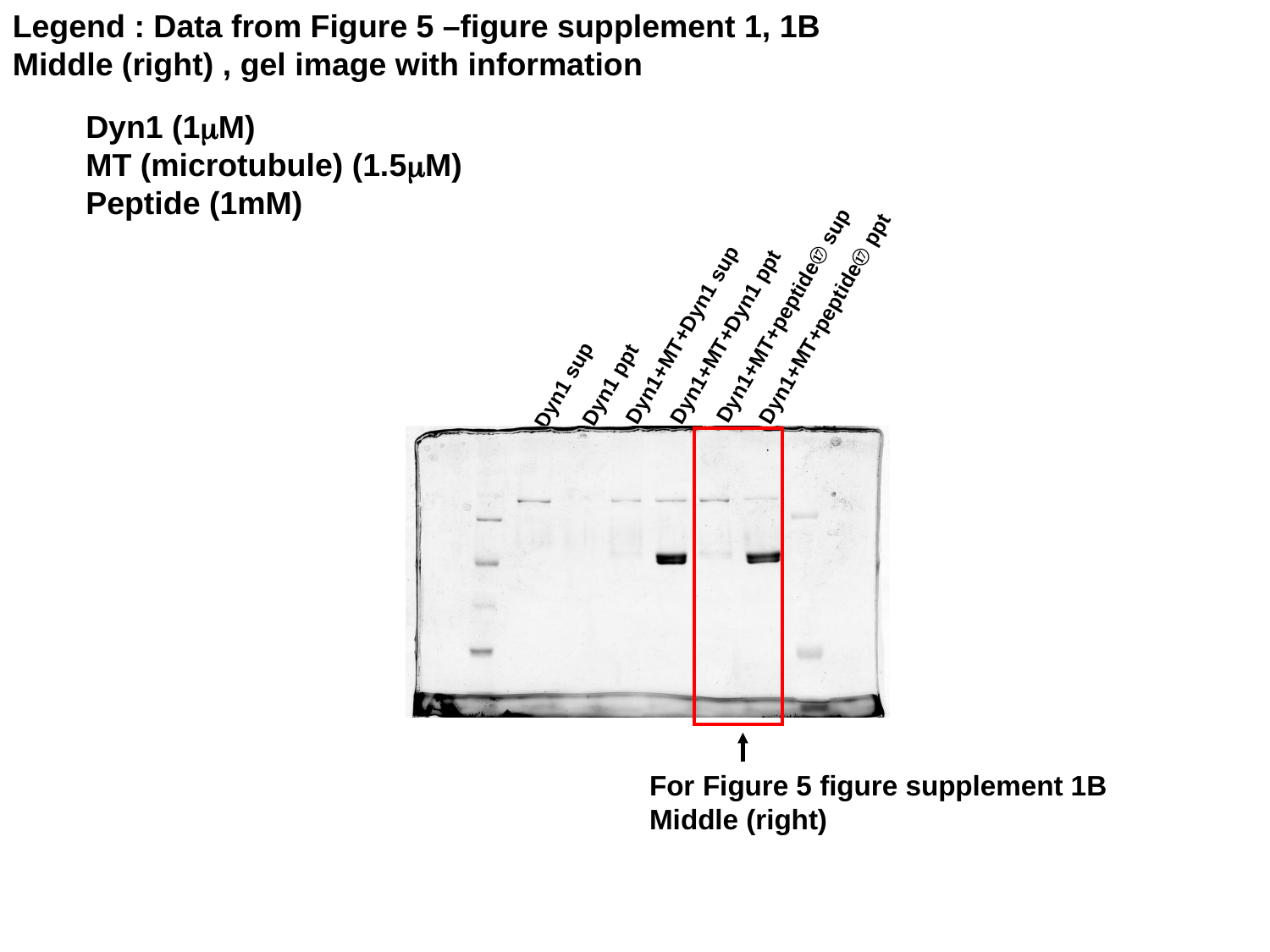

Legend : Data from Figure 5 –figure supplement 1, 1B
Middle (right) , gel image with information
Dyn1 (1mM)
MT (microtubule) (1.5mM)
Peptide (1mM)
Dyn1+MT+peptide⑰ sup
Dyn1+MT+peptide⑰ ppt
Dyn1+MT+Dyn1 sup
Dyn1+MT+Dyn1 ppt
 Dyn1 sup
 Dyn1 ppt
For Figure 5 figure supplement 1B
Middle (right)

## Slide 5
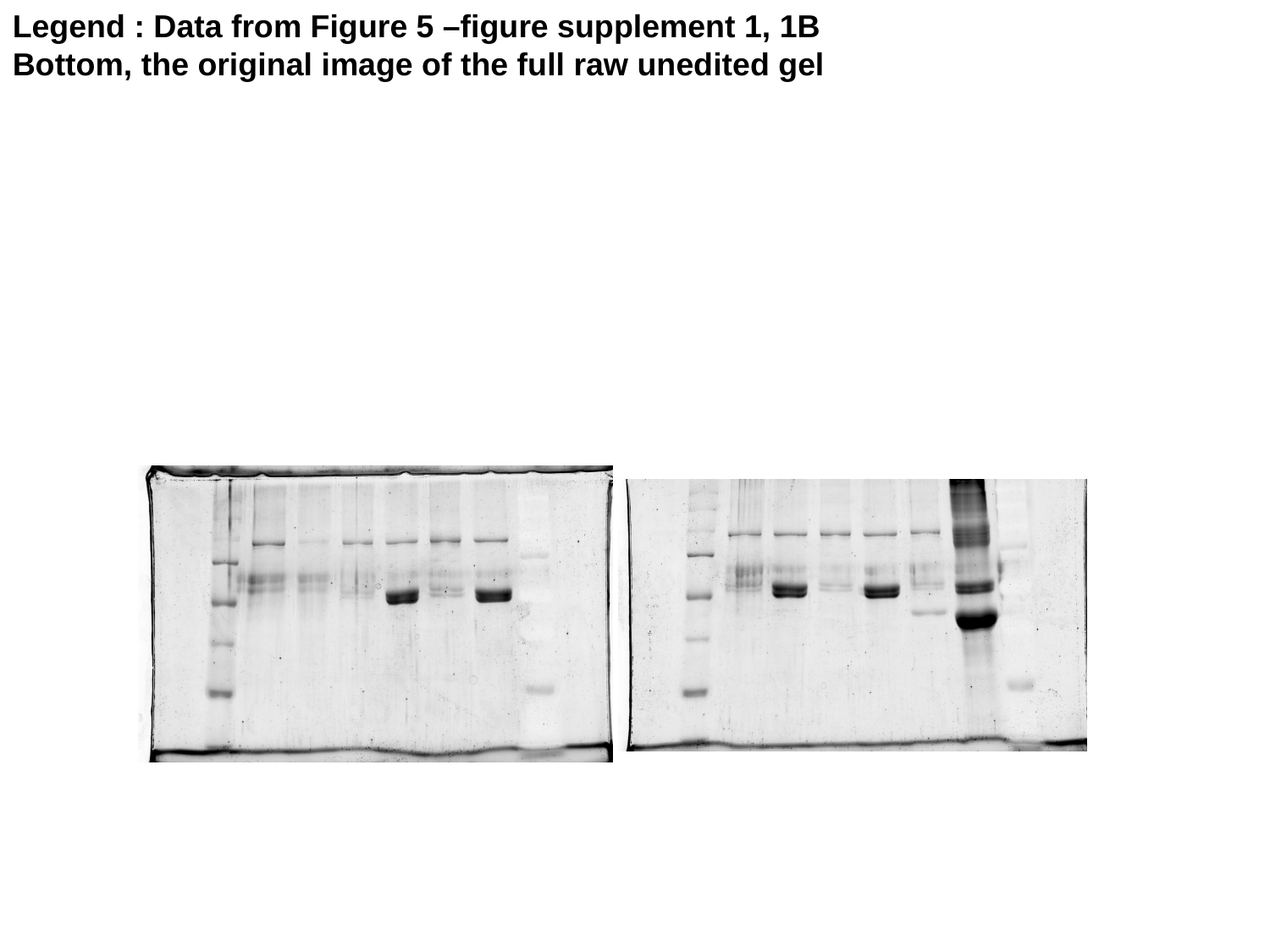

Legend : Data from Figure 5 –figure supplement 1, 1B
Bottom, the original image of the full raw unedited gel

## Slide 6
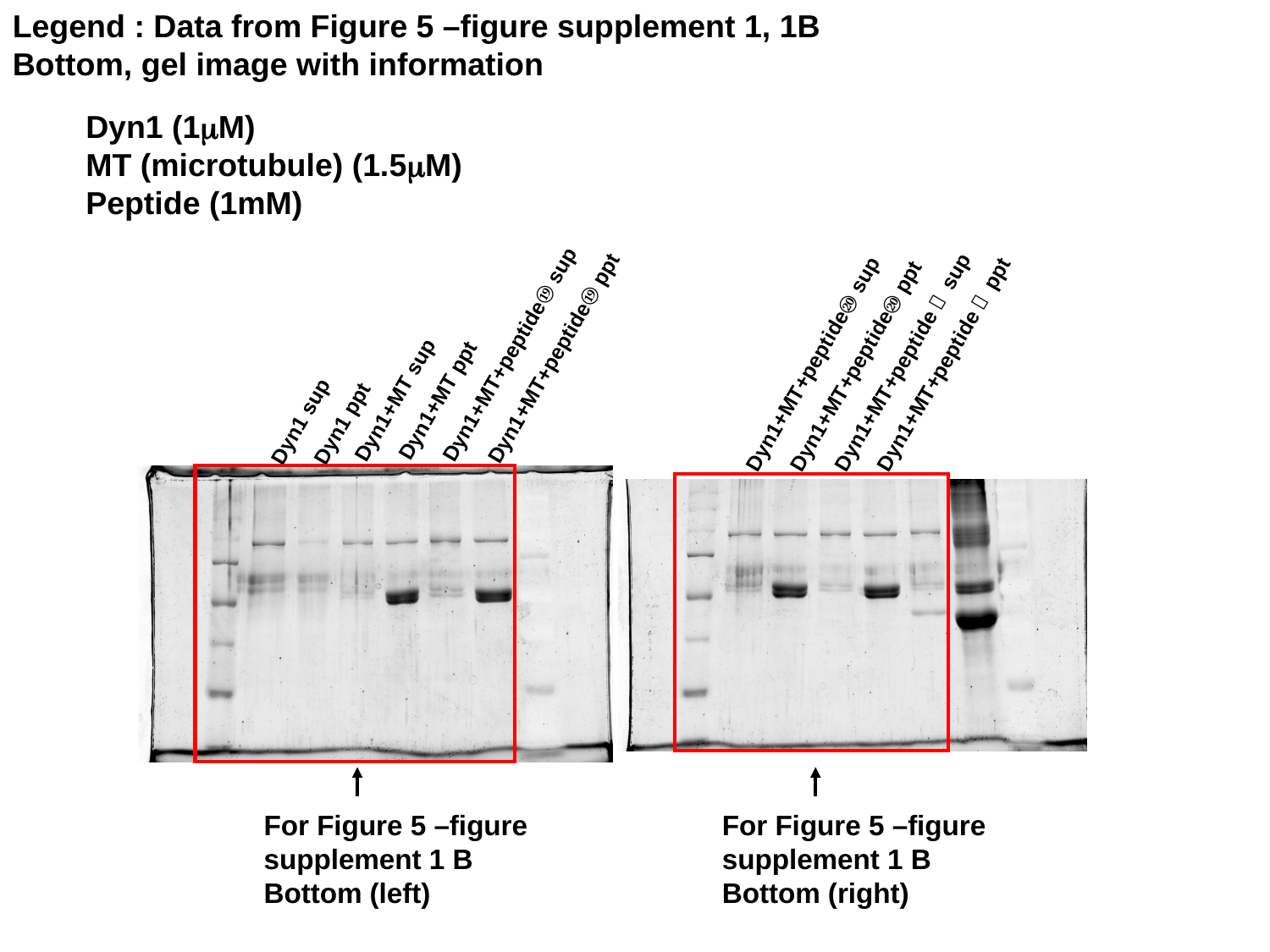

Legend : Data from Figure 5 –figure supplement 1, 1B
Bottom, gel image with information
Dyn1 (1mM)
MT (microtubule) (1.5mM)
Peptide (1mM)
Dyn1+MT+peptide⑲ sup
Dyn1+MT+peptide⑲ ppt
Dyn1+MT+peptide㉑ ppt
Dyn1+MT+peptide⑳ sup
Dyn1+MT+peptide㉑ sup
Dyn1+MT+peptide⑳ ppt
Dyn1+MT ppt
Dyn1+MT sup
 Dyn1 sup
 Dyn1 ppt
For Figure 5 –figure supplement 1 B
Bottom (left)
For Figure 5 –figure supplement 1 B
Bottom (right)

## Slide 7
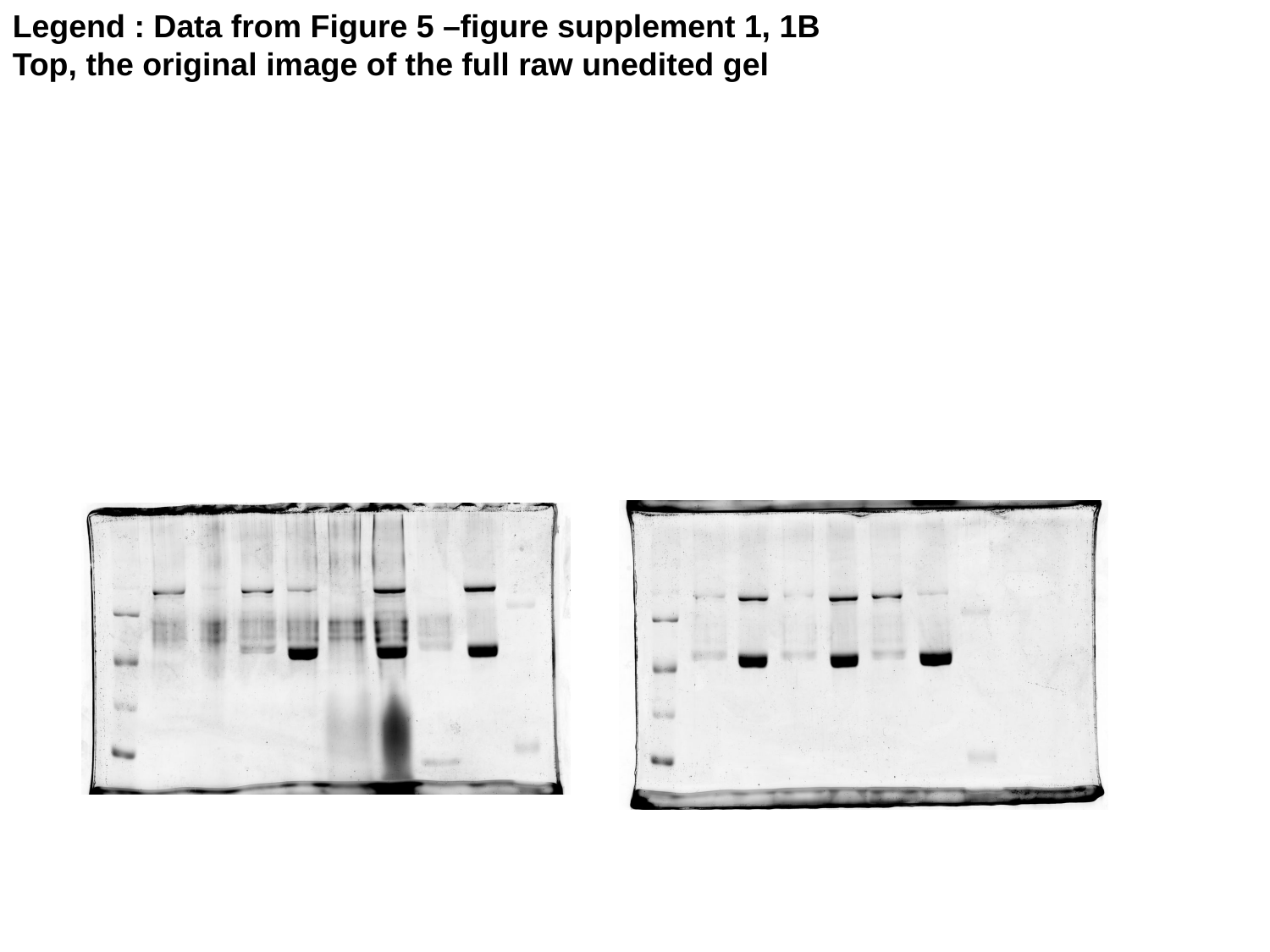

Legend : Data from Figure 5 –figure supplement 1, 1B
Top, the original image of the full raw unedited gel

## Slide 8
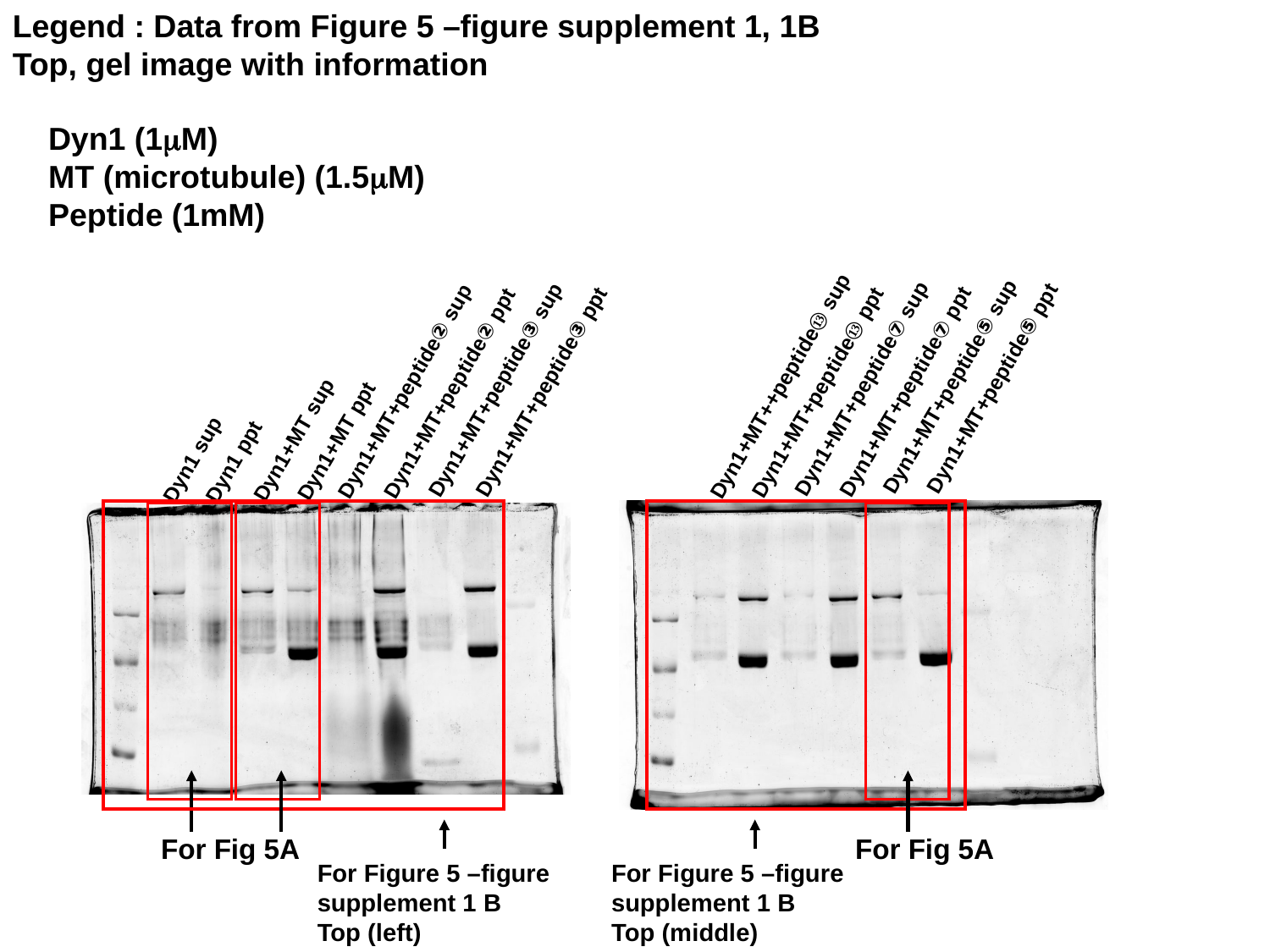

Legend : Data from Figure 5 –figure supplement 1, 1B
Top, gel image with information
Dyn1 (1mM)
MT (microtubule) (1.5mM)
Peptide (1mM)
Dyn1+MT+peptide⑤ ppt
Dyn1+MT+peptide⑤ sup
Dyn1+MT+peptide⑦ sup
Dyn1+MT+peptide⑦ ppt
Dyn1+MT+peptide⑬ ppt
Dyn1+MT++peptide⑬ sup
Dyn1+MT ppt
Dyn1+MT sup
Dyn1+MT+peptide② ppt
Dyn1+MT+peptide③ ppt
Dyn1+MT+peptide② sup
Dyn1+MT+peptide③ sup
 Dyn1 sup
 Dyn1 ppt
For Fig 5A
For Fig 5A
For Figure 5 –figure supplement 1 B
Top (left)
For Figure 5 –figure supplement 1 B
Top (middle)

## Slide 9
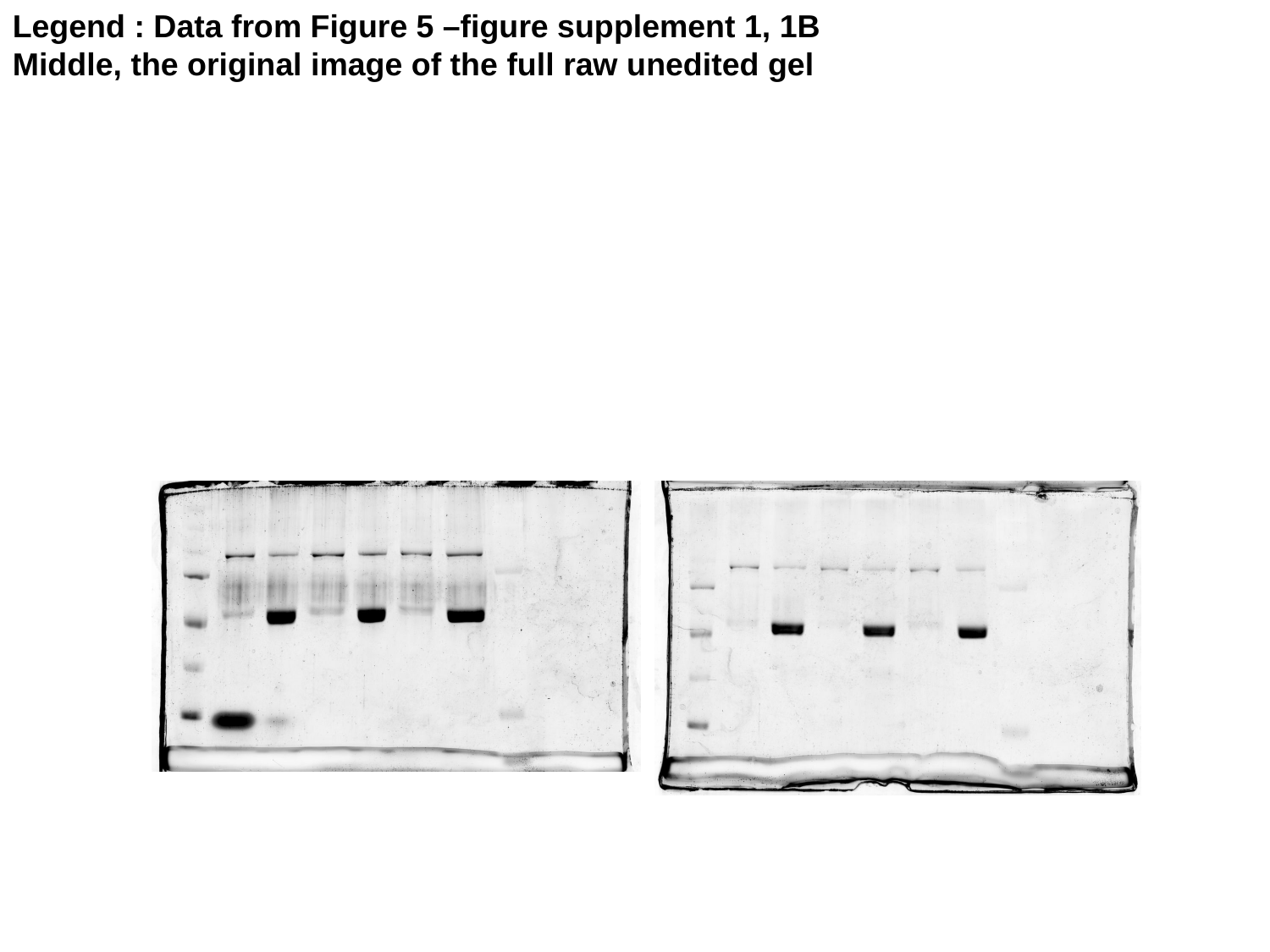

Legend : Data from Figure 5 –figure supplement 1, 1B
Middle, the original image of the full raw unedited gel

## Slide 10
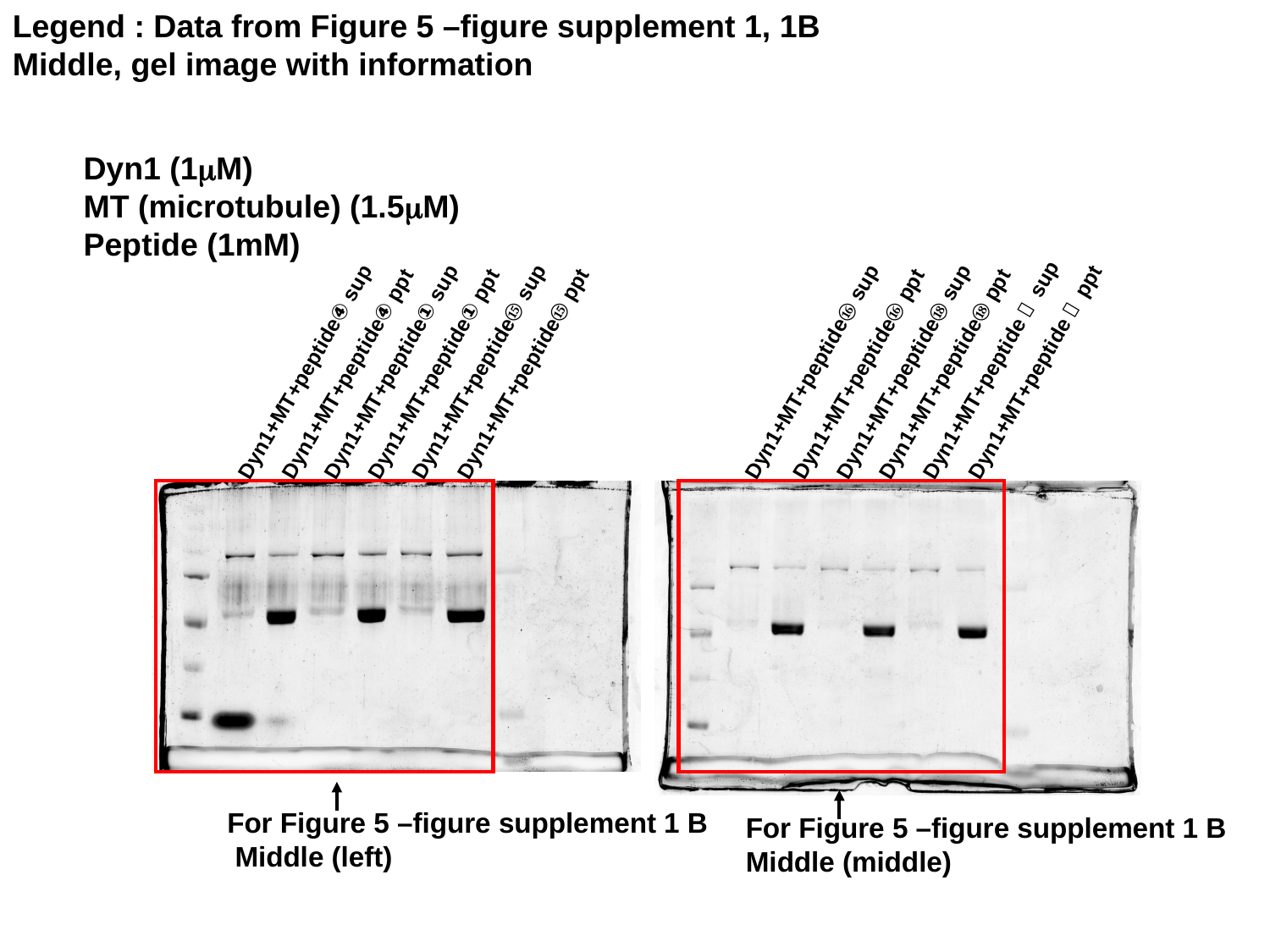

Legend : Data from Figure 5 –figure supplement 1, 1B
Middle, gel image with information
Dyn1+MT+peptide⑮ sup
Dyn1+MT+peptide⑮ ppt
Dyn1+MT+peptide① sup
Dyn1+MT+peptide① ppt
Dyn1+MT+peptide④ sup
Dyn1+MT+peptide④ ppt
Dyn1+MT+peptide㉒ sup
Dyn1+MT+peptide㉒ ppt
Dyn1+MT+peptide⑱ sup
Dyn1+MT+peptide⑱ ppt
Dyn1+MT+peptide⑯ sup
Dyn1+MT+peptide⑯ ppt
For Figure 5 –figure supplement 1 B
 Middle (left)
For Figure 5 –figure supplement 1 B
Middle (middle)
Dyn1 (1mM)
MT (microtubule) (1.5mM)
Peptide (1mM)
