# Supplementary material for: Microtubule assembly by tau impairs endocytosis and neurotransmission via dynamin sequestration in Alzheimer’s disease synapse model
Source: eLife. 2022 Apr 26;11:e73542. doi: 10.7554/eLife.73542 (PMC9071263; doi:10.7554/eLife.73542)
Supplement: Figure 5—figure supplement 2—source data 1. [file elife-73542-fig5-figsupp2-data1.pptx]

## Slide 1
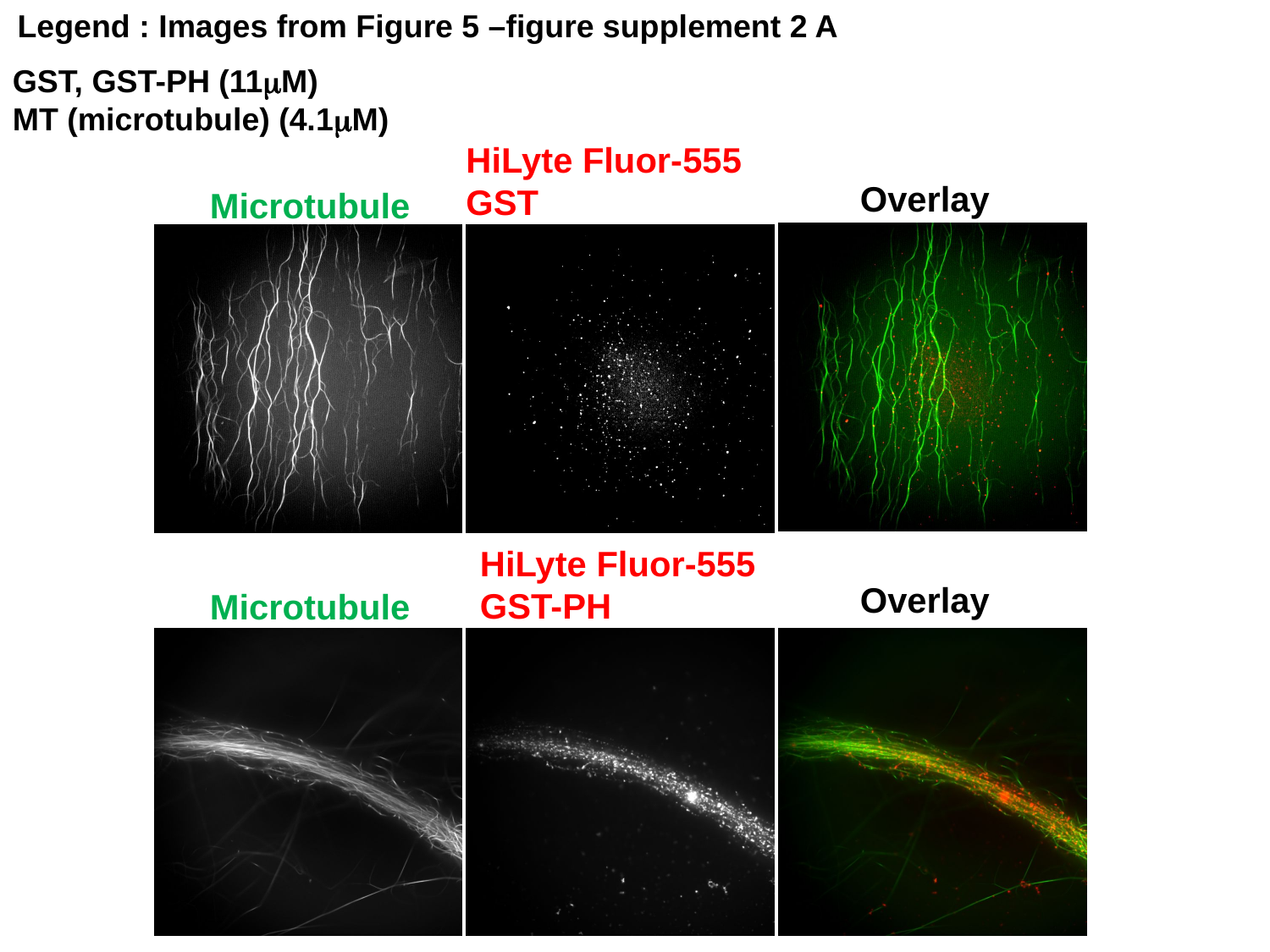

Legend : Images from Figure 5 –figure supplement 2 A
GST, GST-PH (11mM)
MT (microtubule) (4.1mM)
HiLyte Fluor-555
GST
Overlay
Microtubule
HiLyte Fluor-555
GST-PH
Overlay
Microtubule

## Slide 2
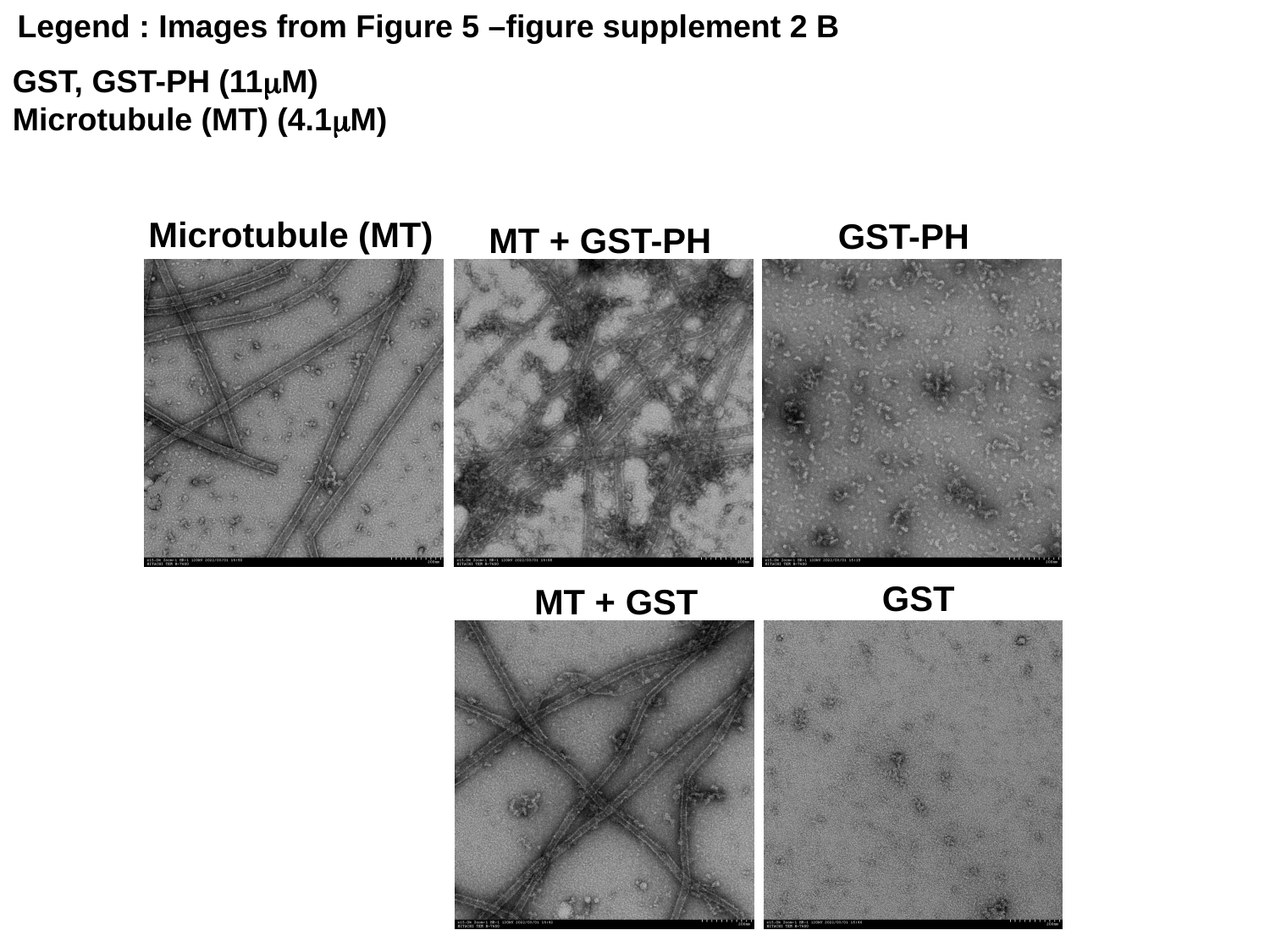

Legend : Images from Figure 5 –figure supplement 2 B
GST, GST-PH (11mM)
Microtubule (MT) (4.1mM)
Microtubule (MT)
GST-PH
MT + GST-PH
GST
MT + GST
